# Diagnostic Accuracy of Deep Learning for Intracranial Hemorrhage Detection in Non-Contrast Brain CT Scans: A Systematic Review and Meta-Analysis

**DOI:** 10.3390/jcm14072377

**Published:** 2025-03-30

**Authors:** Armin Karamian, Ali Seifi

**Affiliations:** 1 School of Medicine, University of Texas Health at San Antonio, San Antonio, TX 78229, USA; karamian.armin91@gmail.com; 2 Division of Neurocritical Care, Department of Neurosurgery, University of Texas Health at San Antonio, San Antonio, TX 78229, USA

**Keywords:** artificial intelligence, deep learning, intracranial hemorrhage, ICH, non-contrast computed tomography, NCCT

## Abstract

**Background**: Intracranial hemorrhage (ICH) is a life-threatening medical condition that needs early detection and treatment. In this systematic review and meta-analysis, we aimed to update our knowledge of the performance of deep learning (DL) models in detecting ICH on non-contrast computed tomography (NCCT). **Methods**: The study protocol was registered with PROSPERO (CRD420250654071). PubMed/MEDLINE and Google Scholar databases and the reference section of included studies were searched for eligible studies. The risk of bias in the included studies was assessed using the QUADAS-2 tool. Required data was collected to calculate pooled sensitivity, specificity, positive predictive value (PPV), and negative predictive value (NPV) with the corresponding 95% CI using the random effects model. **Results**: Seventy-three studies were included in our qualitative synthesis, and fifty-eight studies were selected for our meta-analysis. A pooled sensitivity of 0.92 (95% CI 0.90–0.94) and a pooled specificity of 0.94 (95% CI 0.92–0.95) were achieved. Pooled PPV was 0.84 (95% CI 0.78–0.89) and pooled NPV was 0.97 (95% CI 0.96–0.98). A bivariate model showed a pooled AUC of 0.96 (95% CI 0.95–0.97). **Conclusions**: This meta-analysis demonstrates that DL performs well in detecting ICH from NCCTs, highlighting a promising potential for the use of AI tools in various practice settings. More prospective studies are needed to confirm the potential clinical benefit of implementing DL-based tools and reveal the limitations of such tools for automated ICH detection and their impact on clinical workflow and outcomes of patients.

## 1. Introduction

Intracranial hemorrhage (ICH) is a life-threatening condition and refers to any bleeding within the brain parenchyma and surrounding meningeal spaces [1]. It has been estimated that approximately 2 million individuals are affected by ICH worldwide annually [2]. It is an important cause of death and disability [3]. Early detection and initiation of appropriate treatment are critical to improve outcomes for ICH patients [4]. Non-contrast computed tomography (NCCT) is a standard imaging modality for ICH diagnosis and requires expertise to differentiate between blood and healthy brain parenchyma and assess its extent and location [5,6,7]. However, the diagnosis of ICH in emergency settings may be challenging due to a lack of time, the heavy workload of radiologists, and the shortage of expert radiologists in low-income settings [8,9,10]. While non-radiologist physicians or residents can compensate for these limitations, it may sometimes result in delays in diagnosis or misdiagnosis, potentially leading to life-threatening conditions [11,12,13]. This highlights the necessity for tools to help radiologists effectively and correctly identify ICH.

Artificial intelligence (AI) has, in recent years, had a very significant impact on most aspects of healthcare [14,15]. AI has the potential to improve radiologist efficiency, optimize workflow, and standardize ICH detection. Deep learning (DL), a subfield of machine learning (ML) and AI that utilizes artificial neural networks (ANN), possesses the capability to learn from data, making it a potent tool in computing with wide applications spanning numerous domains, including healthcare, visual recognition, and many others [16,17]. Unlike traditional machine learning, DL is able to learn the most critical features of a given task and perform tasks such as classification, detection, and segmentation on medical images simultaneously [18,19,20]. Convolutional Neural Network (CNN) is a specialized type of DL algorithm that has become popular in many computer-vision tasks and has recently gained attention in the health service area such as radiology. It uses convolutional layers that effectively extract local features from medical images [21,22,23].

Several previous studies have reported the efficacy and high accuracy of DL in the automated detection of ICH on non-contrast brain CT scans, which encourages the use of DL in clinical practice [20,24,25,26,27]. Regarding processing time, DL models have shown promising results, achieving a quicker first diagnosis compared to the time taken by radiologists to make their initial diagnoses [28]. Furthermore, it has been demonstrated that implementing DL for ICH detection from NCCT significantly reduced costs and shortened the length of emergency department stay [29]. Thus, it has the potential to help and improve the performance of radiologists in evaluating NCCT scans suspicious for ICH, especially in emergency scenarios, when time efficiency is required. By reducing the increasing workload, radiologists can devote their attention to more complex and challenging cases. Consequently, in this systematic review and meta-analysis, we aimed to update our knowledge of the per-scan diagnostic performance of DL-based models in detecting ICH.

## 2. Materials and Methods

This systematic review and meta-analysis was registered with PROSPERO (CRD420250654071) and written following the Preferred Reporting Items for Systematic Reviews and Meta-Analyses (PRISMA) and the Preferred Reporting Items for Systematic Review and Meta-analysis of Diagnostic Test Accuracy Studies (PRISMA-DTA) guidelines.

### 2.1. Search Strategy and Study Selection

The literature search was conducted in two public databases (PubMed/MEDLINE and Google Scholar) from inception to 23 February 2025, using combinations of the following keywords and MeSH terms: “Intracranial hemorrhage”, “Intracranial haemorrhage”, “ICH”, “Artificial Intelligence”, “AI”, “Machine Learning”, and “Deep Learning”. Two independent reviewers (A.K. and A.S.) screened the initial search to identify all eligible studies. Any disagreements between reviewers were resolved by reaching a consensus during the discussion. The reference lists of the included studies were also manually screened for additional pertinent articles.

We included studies that met the following criteria: (1) used a deep learning model for the detection of ICH of any type (intraparenchymal hemorrhage (IPH), subdural hemorrhage (SDH), epidural hemorrhage (EDH), intraventricular hemorrhage (IVH), and subarachnoid hemorrhage (SAH)) on NCCT scans, (2) published in peer-reviewed journals, (3) Full-text is available, (4) published in the English language, and (5) reported data on the diagnostic accuracy of the DL-based algorithms. Non-English manuscripts, case reports, case series, abstracts, preprints, conference papers, editorials, commentaries, book chapters, review articles, and studies that used non-deep learning methods were excluded. We also excluded studies that did not provide sufficient data on the diagnostic accuracy of DL-based models for ICH detection on NCCT scans.

### 2.2. Data Extraction

We extracted data from selected studies including first author, year of publication, study design, type of used databases for training and testing the DL model, size of the databases, type of DL model, gold standard, and performance results including accuracy, specificity, sensitivity, and Area Under the Curve (AUC). The true positive (TP), false positive (FP), true negative (TN), and false negative (FN) were also extracted, and if not reported, were calculated based on the provided values for sensitivity, specificity, and the total number of positive or negative NCCTs.

### 2.3. Risk of Bias Assessment

All included studies were evaluated for the potential risk of bias using a revised tool for quality assessment of diagnostic accuracy studies included in the systematic review (QUADAS-2) [30]. This tool comprises four domains: patient selection, index test, reference standard, and flow and timing.

### 2.4. Statistical Analysis

Statistical analysis in R v4.3.3 was conducted with the “meta” and “mada” packages. Values of TP, TN, FP, and FN were collected to compute pooled sensitivity, specificity, positive predictive value (PPV), and negative predictive value (NPV) with the corresponding 95% CI using random effects model. If in a study more than one DL model were compared, we used the performance results of the best-performing model. A summary receiver operating characteristic (SROC) curve was generated by the bivariate model using the “reitsma” function of the “mada” package. The AUC, which measures the entire two-dimensional area underneath the ROC curve, was also calculated to show the ability of DL models to identify ICH on NCCTs (the AUC of 1.0 indicates perfect discrimination, while an AUC of 0.5 indicates random chance).

Tau^2^ and I^2^ statistics were used to evaluate statistical heterogeneity testing. The heterogeneity was classified as low (0% to 25%), moderate (25% to 50%), or high (≥50%) based on the calculated I^2^ [31]. Funnel plots were used to visualize publication bias and Egger’s regression test was performed to estimate publication bias in included studies. A *p*-value less than 0.05 was considered a significant publication bias.

## 3. Results

**Figure 1:**
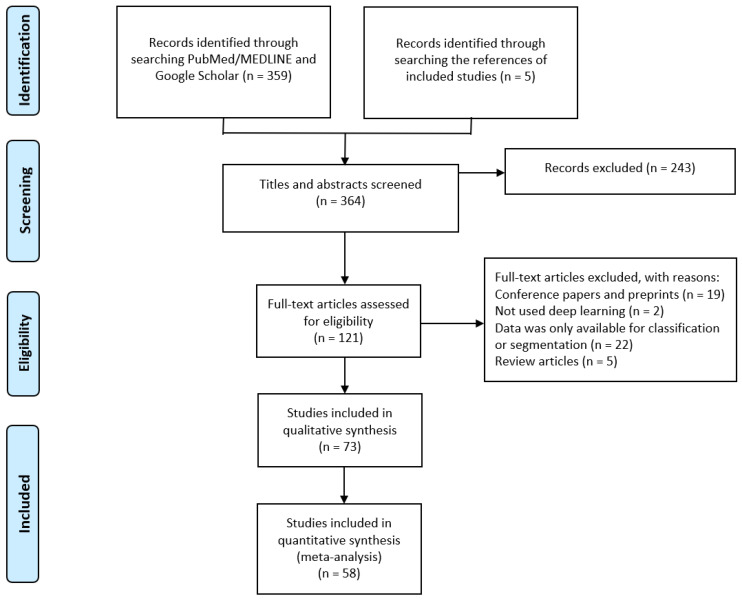
PRISMA flow diagram.

The primary search on databases resulted in 359 articles. Five eligible studies were also obtained by searching the reference section of the selected studies. In total, 121 full-text articles were reviewed for eligibility. At last, seventy-three studies were included in our qualitative synthesis [7,19,20,24,25,26,27,28,29,32,33,34,35,36,37,38,39,40,41,42,43,44,45,46,47,48,49,50,51,52,53,54,55,56,57,58,59,60,61,62,63,64,65,66,67,68,69,70,71,72,73,74,75,76,77,78,79,80,81,82,83,84,85,86,87,88,89,90,91,92,93,94,95]. Given that in 15 studies the relevant data for calculating values of TP, TN, FP, and FN was unavailable, fifty-eight studies were selected for our meta-analysis (Figure 1). A summary of the characteristics of the included studies is provided in Table 1. Sixty-one studies were retrospective and nine were prospective studies. Three studies had a retrospective/prospective design.

The results of the risk of bias assessment are shown in Table S1. Domains related to patient selection and reference standard were associated with increased risk of bias. 

**Figure 2:**
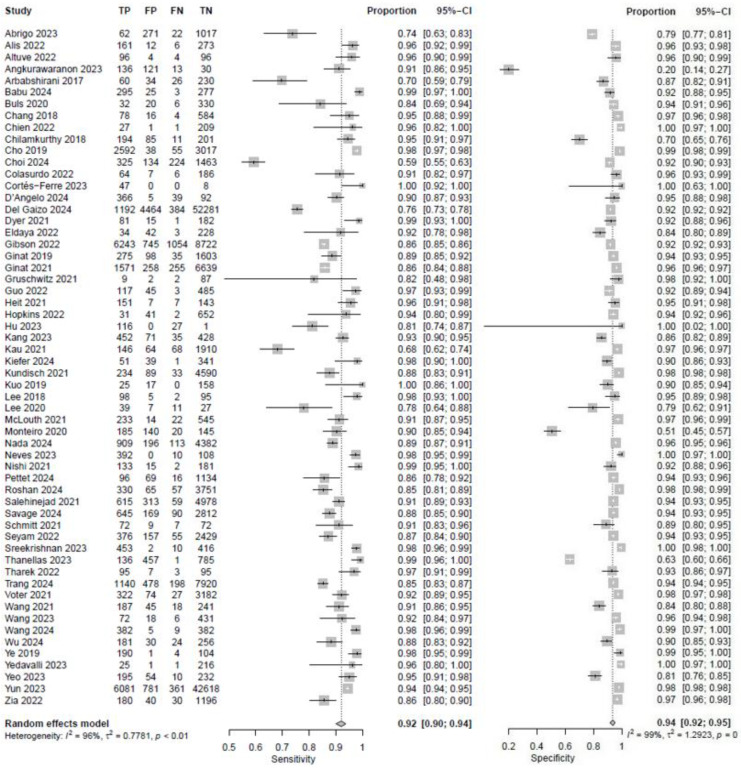
Sensitivity and specificity of deep learning models in detecting intracranial hemorrhage on non-contrast computed tomography in included studies [7,19,20,24,25,27,28,29,32,33,35,36,37,38,39,40,42,45,46,47,48,49,50,51,53,55,56,57,58,59,60,61,62,65,66,67,68,69,72,73,74,75,76,78,80,81,82,84,85,86,87,88,89,90,91,92,94].

**Figure 3:**
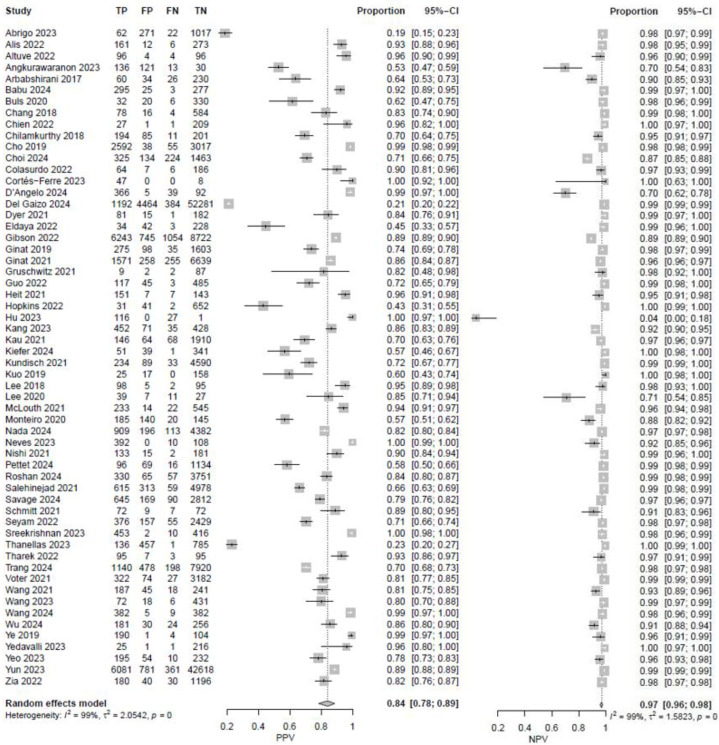
Positive and negative predictive values of deep learning models in detecting intracranial hemorrhage on non-contrast computed tomography in included studies [7,19,20,24,25,27,28,29,32,33,35,36,37,38,39,40,42,45,46,47,48,49,50,51,53,55,56,57,58,59,60,61,62,65,66,67,68,69,72,73,74,75,76,78,80,81,82,84,85,86,87,88,89,90,91,92,94].

**Figure 4:**
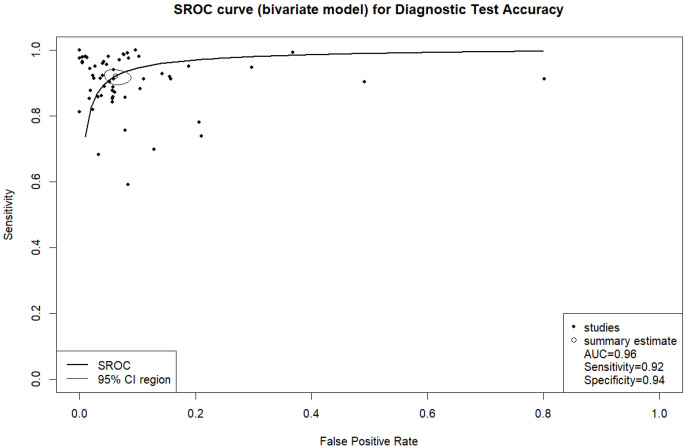
Bivariate summary receiver operating characteristic (SROC) curve of deep learning models for detecting intracranial hemorrhage on non-contrast computed tomography in included studies.

In fifty-eight studies, the values for TP, TN, FP, and FN were reported or corresponding data was available to calculate these numbers for the calculation of pooled sensitivity, specificity, PPV, and NPV. A pooled sensitivity of 0.92 (95% CI 0.90–0.94, I^2^ = 96%) and a pooled specificity of 0.94 (95% CI 0.92–0.95, I^2^ = 99%) were achieved (Figure 2). Pooled PPV was 0.84 (95% CI 0.78–0.89, I^2^ = 99%), and pooled NPV was 0.97 (95% CI 0.96–0.98, I^2^ = 99%) (Figure 3). A bivariate model for the diagnostic accuracy of DL in the detection of ICH on NCCTs was performed (Figure 4). In this model, AUC was 0.96 (95% CI 0.95–0.97).

### 3.1. Subgroup Analyses

**Figure 5:**
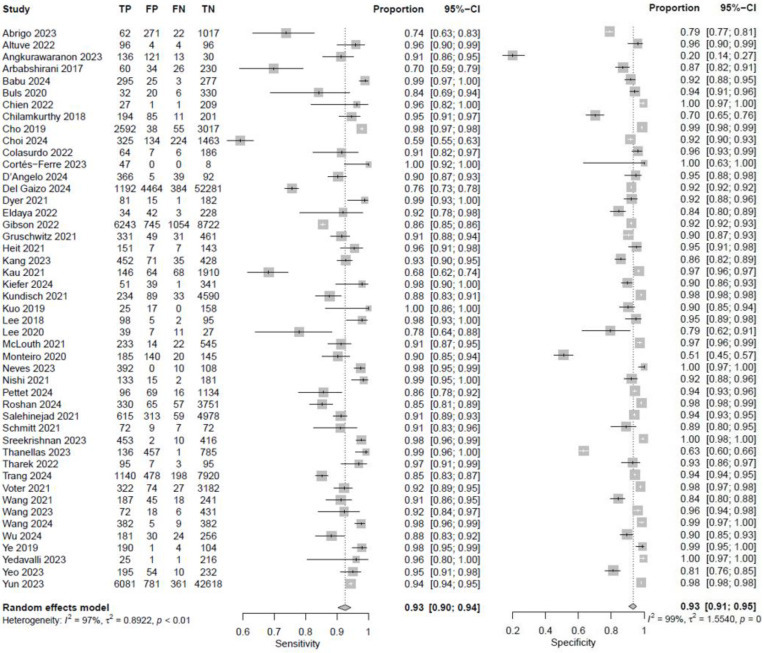
Sensitivity and specificity of deep learning models in detecting intracranial hemorrhage on non-contrast computed tomography according to retrospective studies [7,19,24,25,27,28,29,32,33,35,36,38,39,40,42,45,46,47,53,56,57,58,59,60,61,62,65,66,68,69,72,73,75,78,80,81,82,84,85,86,87,88,89,90,91,92].

**Figure 6:**
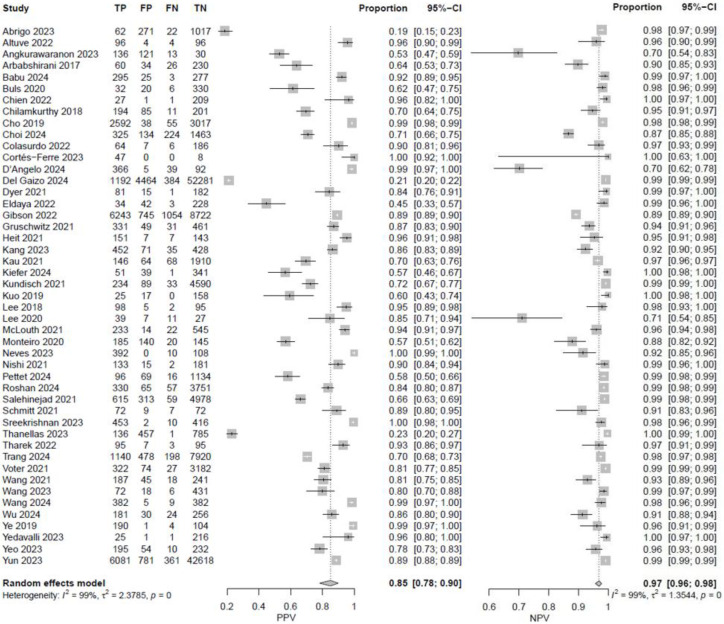
Positive and negative predictive values of deep learning models in detecting intracranial hemorrhage on non-contrast computed tomography according to retrospective studies [7,19,24,25,27,28,29,32,33,35,36,38,39,40,42,45,46,47,53,56,57,58,59,60,61,62,65,66,68,69,72,73,75,78,80,81,82,84,85,86,87,88,89,90,91,92].

**Figure 7:**
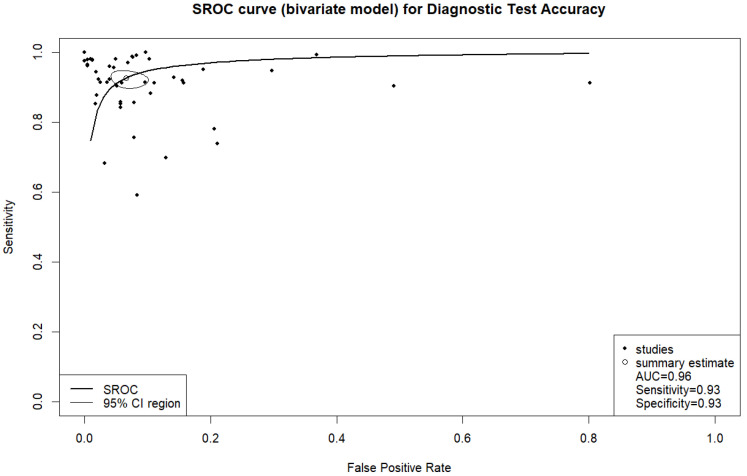
Bivariate summary receiver operating characteristic (SROC) curve of deep learning models for detecting intracranial hemorrhage on non-contrast computed tomography according to retrospective studies.

We performed further analyses based on the study design. Forty-seven retrospective studies provided data for calculating the pooled diagnostic performance of DL models in ICH detection from NCCTs. The pooled sensitivity and specificity were 0.93 (95% CI 0.90–0.94, I^2^ = 97%) and 0.93 (95% CI 0.91–0.95, I^2^ = 99%), respectively (Figure 5). Pooled PPV was 0.85 (95% CI 0.78–0.90, I^2^ = 99%), and pooled NPV was 0.97 (95% CI 0.96–0.98, I^2^ = 99%) (Figure 6). The bivariate model based on the results from retrospective studies showed an AUC of 0.96 (95% CI 0.94–0.98) (Figure 7).

**Figure 8:**
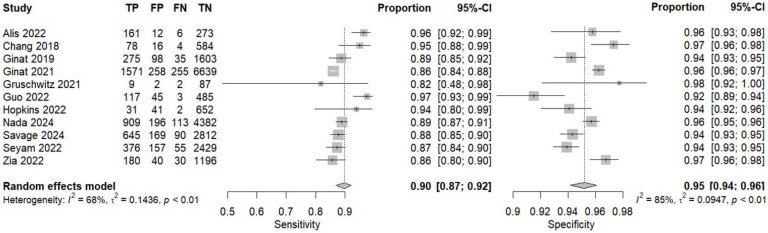
Sensitivity and specificity of deep learning models in detecting intracranial hemorrhage on non-contrast computed tomography according to prospective studies [20,37,48,49,50,51,55,67,74,76,94].

**Figure 9:**
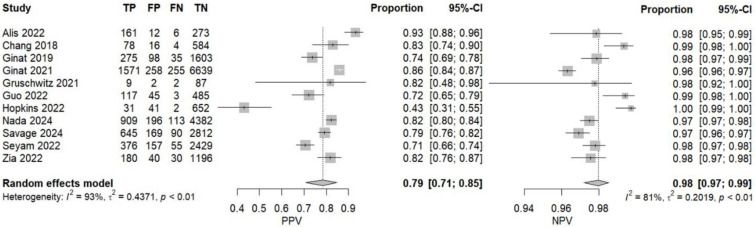
Positive and negative predictive values of deep learning models in detecting intracranial hemorrhage on non-contrast computed tomography according to prospective studies [20,37,48,49,50,51,55,67,74,76,94].

**Figure 10:**
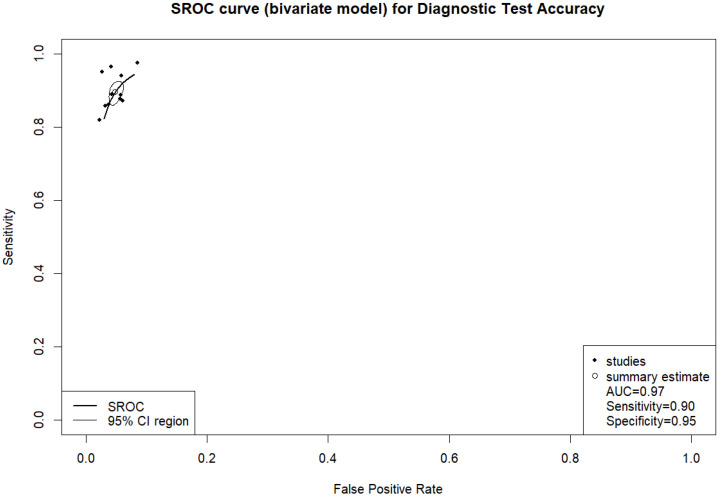
Bivariate summary receiver operating characteristic (SROC) curve of deep learning models for detecting intracranial hemorrhage on non-contrast computed tomography according to prospective studies.

According to the data from eleven prospective studies, a pooled sensitivity of 0.90 (95% CI 0.87–0.92, I^2^ = 68%) and a pooled specificity of 0.95 (95% CI 0.94–0.96, I^2^ = 85%) were achieved (Figure 8). Pooled PPV and pooled NPV were 0.79 (95% CI 0.71–0.85, I^2^ = 93%) and 0.98 (95% CI 0.97–0.99, I^2^ = 81%), respectively (Figure 9). The bivariate model based on the results from prospective studies showed an AUC of 0.97 (95% CI 0.95–0.98) (Figure 10).

### 3.2. Publication Bias

Funnel plots and Egger’s regression test were used to estimate publication bias in included studies (Figure S1a–d). Eggers’ test does not indicate the presence of funnel plot asymmetry in pooled specificity (*p*-value 0.9446), pooled PPV (*p*-value 0.0976), and pooled NPV (*p*-value 0.4335); however, it shows funnel plot asymmetry in pooled sensitivity (*p*-value 0.0135).

## 4. Discussion

ICH is a life-threatening condition, associated with a very high morbidity and mortality rate; thus, early diagnosis and treatment are critical to improve the prognosis of patients [96,97]. AI has the potential to help radiologists in ICH detection from NCCTs and enhance clinical workflow [46,59,76,98,99]. DL models can learn to identify subtle features associated with ICH that are difficult for radiologists to detect, thus saving time and directing radiologists’ attention to the most critical cases. In emergency settings, identifying bleeding as a binary classification is crucial in prompt intervention’s first step. In one study, AI-assisted re-evaluation of head NCCTs improved the mean accuracy of board-certified emergency physicians in identifying ICHs from 87.70% to 93.85% [98], which shows the potential usefulness of AI tools in reducing missed positive cases by radiologists in clinical practice. This systematic review and meta-analysis aimed to determine the diagnostic accuracy of DL models in detecting ICH (presence or absence) on NCCTs. The results showed that DL has a promising diagnostic performance in the automated detection of ICH from NCCTs with a high sensitivity and specificity of 0.92 and 0.94, respectively. The PPV, NPV, and AUC were also found to be 0.84, 0.97, and 0.96, respectively.

A DL model that has more sensitivity and fewer false positives can greatly reduce the workload of radiologists. Although the goal is to increase the sensitivity for the detection of true positive cases as much as possible, the feature-based balance, which is related to the detection of true negatives, should be considered the ultimate goal for an ideal DL model in emergency settings. Dyer et al. developed a DL model with EfficientNet neural network architecture for triaging patients with head CT abnormalities. The model was validated on a multi-center dataset and reached a sensitivity of 98.8% and a specificity of 92.5% for ICH detection from NCCTs [45]. Cho et al. proposed a DL model using a cascade of CNNs and dual fully convolutional networks (FCNs) for detecting the presence or absence of bleeding on NCCTs. In this model, the sensitivity and specificity were found to be 97.91% and 98.76%, respectively.

In recent years, several commercial AI tools have been developed to identify ICH in NCCTs. Aidoc is a commercially available, FDA- and CE-cleared CNN-based AI tool for detecting ICH from NCCTs. It has been trained and tested on CT scans from 9 medical centers and 17 different CT machines [57]. In a study by Buls et al., the Aidoc tool was used to detect ICH cases from a local dataset consisting of 388 NCCTs. The results showed a sensitivity of 84% and a specificity of 0.94% [36]. In another study, Ginat used Aidoc as a tool for prioritizing positive ICH cases on NCCTs. This study used a large local dataset of 8723 NCCTs from a single tertiary care center. The AI tool reached a sensitivity and specificity of 88.4% and 96.1%, respectively [49]. However, due to its single-center design, the findings may not apply to other practice settings. Conversely, in another study by Kau et al., the diagnostic accuracies of a second-year resident and the Aidoc differed highly significantly (*p* < 0.001) and both resident and neuroradiologist under time pressure outperformed the Aidoc in ICH detection from NCCTs [57].

RAPID ICH is another commercial AI tool that uses a hybrid deep 2D–3D deep CNN structure for detecting ICH on NCCTs [46,53]. Testing this AI tool on a single-center small dataset showed acceptable sensitivity, specificity, and NPV of 91.9%, 84.4%, and 98.7%, respectively, but a low PPV of 44.7%. In this study, the most common causes of high false-positive cases were motion, streak artifacts, calcifications, and dural thickening [46]. Another study by Heit et al. assessed the performance of the RAPID ICH tool for ICH detection from NCCTs. Testing the AI tool on a local independent dataset from the training dataset showed a sensitivity of 95.6% and a specificity of 95.3%. The tool also achieved a high PPV and NPV of 95.6% and 95.3%, respectively, for ICH detection [53].

Recently, another AI application called Viz.ai ICH has been developed to enhance and expedite the diagnosis of suspected ICH cases from NCCTs. Analyzing 4203 NCCTs, compared to the gold standard of the neuroradiologist’s impression, Viz.ai ICH demonstrated a sensitivity of 85% and a specificity of 98% enhancing radiologists’ workflow by correctly detecting NCCTs without bleeding as negative for ICH. For ICHs with larger volume/size, the sensitivity improved to 100% [72].

Another study by McLouth et al. validated a commercially available DL-based tool called CINA v1.0 device for detecting ICH [65]. The tool showed high sensitivity, specificity, and accuracy of 91.4%, 97.5%, and 95.6%, respectively, for ICH detection. The sensitivity reached 100% for medium (5–25 mL) and large (>25 mL) ICH. Del Gaizo tested the performance of the CINA v1.0 device on a large dataset for detecting ICH. The AI tool yielded reduced sensitivity and specificity compared with the published literature with a sensitivity of 75.6%, specificity of 92.1%, and accuracy of 91.7%. The authors concluded that the performance of this application for ICH detection was low in a low-prevalence environment (2.7%) because of the increased median time that the radiologists spent on reading false-positive and false-negative findings (1 min 14 s and 1 min 5 s, respectively), potentially reducing system efficiency [44]. This highlights the importance of testing AI tools in different clinical settings to achieve a better institutional decision-making system.

Since distinguishing ICH from bony adjacent regions on CT scans may be challenging for radiologists [78,100], applying image processing techniques by removing skull and facial bones will help retain only the intracranial structures and reduce the misdetection of ICH cases [60]. The present models have been specifically trained for ICH detection and not trained for the detection of other intracranial pathologies such as tumors, hydrocephalus, intracranial calcifications, and ischemic infarcts [32,33,39,95], resulting in false positive cases which may be seen as a drawback of the existing DL models. Another limitation in detecting ICH with AI tools is the difficulty of differentiating between normal brain tissue and some specific subtypes of ICH such as SDH in the subacute phase and small SAHs when mixed with cerebrospinal fluid (CSF) [56,76].

### 4.1. Limitations

This study has some limitations. Most of the included studies had a retrospective design, highlighting the gap in prospectively evaluating DL-based models in real-world clinical settings. The diversity of CT scanners, vendors, gold standards, and datasets utilized for training and validating DL methods makes it difficult to interpret the combined performance results. 

Most studies did not provide sufficient separate data on the diagnostic performance of DL models for detecting different ICH subtypes (e.g., SAH, SDH, IVH, etc.). This could be a topic for future research to create datasets that include only specific ICH subtype cases to assess the diagnostic performance of DL models for each subtype. Additionally, there is limited information on the diagnostic performance of DL models concerning hemorrhage volume. This factor is critical, as smaller hemorrhages are more likely to be overlooked in emergency situations compared to larger, diffuse intracerebral hemorrhages. Because of that, in certain scenarios, subtle bleeding can be clinically significant (e.g., minor SDHs in anticoagulated patients or subtle cisternal aneurysmal SAH) and may benefit from assistance by DL algorithms. Moreover, there remains insufficient data on whether utilizing AI tools independently or as assistance for radiologists reduces the interpretation time and workload of radiologists. Furthermore, more studies are needed to assess the cost-effectiveness of using AI solutions as a diagnostic tool in clinical settings. 

### 4.2. Ethical Implications and Responsibilities

Integrating DL models into radiological diagnostics offers many potential benefits, but also carries significant ethical implications and responsibilities. Strict adherence to data privacy regulations and robust cybersecurity measures must be implemented. There may be bias in training datasets; therefore, efforts should be made to make diverse and representative datasets and the benefits of using DL in radiology available to all populations, regardless of socioeconomic status or geographic location. DL models can be complex, making it difficult to understand how they arrive at their decisions, which raises concerns about their accountability and trust; thus, efforts to improve their transparency and explainability are needed. Radiologists must maintain their critical thinking skills and not blindly rely on reports generated by AI. In emergency scenarios when an AI model makes a mistake, determining liability can be problematic; therefore, clear guidelines and legal frameworks are needed. We need to validate and rigorously test DL models before applying them to clinical practice.

### 4.3. Directions for Future

AI tools can aid radiologists in screening CT scans, helping them focus on complex cases. In the future, they are anticipated to play an important role in the education of radiology residents. Where resources and expertise are limited, AI solutions can help diagnose abnormalities in CTs. Considering that less experienced radiologists might miss subtle hemorrhages, future studies should aim to compare the reports produced by the AI tools with those from radiologists to estimate false positives or false negatives accurately. Additionally, DL algorithms can standardize the interpretation of CT scans, reducing inter-observer variability in diagnosing ICH from NCCTs. In clinical settings, AI applications can act as a real-time second reader for images reported as normal, making sure that no positive cases are overlooked. Obtaining larger, more diverse, and accurately annotated datasets for training may increase the generalizability and reproducibility of DL models. Lastly, DL algorithm codes ought to be accessible publicly to researchers in the field so they can enhance and refine their models. Additional prospective multicenter studies with larger datasets are needed to confirm the efficacy of DL-based tools in identifying ICH in NCCTs. 

## 5. Conclusions

This meta-analysis showed that the DL-based models have acceptable diagnostic performance for detecting ICH from NCCTs, underscoring a promising potential for the use of AI tools in various practice settings. More prospective studies are needed to confirm the potential clinical benefit of implementing DL-based tools for automated ICH detection and their impact on clinical workflow and patient outcomes, especially mortality.

## Figures and Tables

**Table 1:** Characteristics of included studies.

Study	Design	Type of ICH	Datasets	DL Model	Ground Truth	Sensitivity (%)	Specificity (%)	Accuracy (%)	AUC	Comments on Performance
Abrigo 2023 [32]	Retrospective	ICH	Trained on RSNA public dataset/tested on a local dataset consisting of 1372 NCCTs	CNN	Radiologists or senior radiology trainees	73.8	79	78.6	0.842	Detection/per-scan
Alis 2022 [20]	Prospective	ICH	Local datasets/trained on 49,968 NCCTs/tested on 452 NCCTs	Joint CNN-RNN	Experienced neuroradiologists	96.41	95.79	96.02	0.961	Detection/per-scan
Altuve 2022 [24]	Retrospective	ICH	Publicly available Kaggle’s Head CT-Hemorrhage database including 200 NCCTs	ResNet-18 (deep residual CNN)	NA	95.65	96.2	95.93	NA	Detection/per-scan/the average performance is reported
Angkurawaranon 2023 [19]	Retrospective	ICH	Local dataset including 300 NCCTs	DL	Experienced neuroradiologists	82	90	89	NA	Detection of ICH/location-level performance is reported
Arbabshirani 2017 [33]	Retrospective	ICH	Local dataset/trained on 46,583 NCCTs/tested on 347 NCCTs	CNN with 3D-architecture	Experienced neuroradiologists	70	87	84	NA	Detection/per-scan
Arman 2023 [34]	Retrospective	ICH	Trained and tested on NCCTs from RSNA public dataset	DenseNet	Neuroradiologists	84.32	98.33	96.32	NA	Detection/per-slice
Babu 2024 [35]	Retrospective	ICH	Publicly available Kaggle dataset/1600 NCCTs were used for training, 600 images for testing, and 400 images for validation	Hybrid DenseNet 121 + LSTM models	Expert radiologists	98.99	NA	97.5	NA	Detection/per-scan
Bark 2024 [95]	Retrospective	ICH	Trained on a dataset of around 100,000 scans/tested on a local dataset of 2306 head CTs	HealthICH+ version 3.1.24 (3D CNN)	Neuroradiologist	NA	NA	NA	NA	Detection/per-scan/PPV of 82.3%
Buls 2020 [36]	Retrospective	ICH	Local dataset/tested on 388 NCCTs	Aidoc (CNN)	Board certified experienced neuroradiologists	84	94	93	NA	Detection/per-scan
Chang 2018 [37]	Prospective	ICH	Trained on a local dataset of 10,159 NCCTs/tested on a local dataset including 682 NCCTs	Hybrid 3D/2D mask ROI-based CNN	Board-certified radiologist	95.1	97.3	97	0.981	Detection/per-scan The accuracy for ICHs > 25, 5–25, 0.01–5.0, and <0.01 mL is 0.997, 0.977, 0.906, and 0.872, respectively
Chien 2022 [29]	Retrospective	ICH	Local dataset/tested on 238 NCCTs	Deep-CNN	NA	96.43	99.52	99.16	NA	Detection/per-scan
Chilamkurthy 2018 [38]	Retrospective	ICH	Trained on a local dataset (Qure25k dataset) including 21,095 NCCTs/tested on a local dataset (CQ500) including 491 NCCTs	Modified ResNet18 (CNN)	Experienced radiologists	94.7	70.1	NA	0.94	Detection/per-scan
Cho 2019 [25]	Retrospective	ICH	Trained on a local dataset of 5702 NCCTs	Cascade of CNNs and dual FCNs	A team of experienced neurologist, neurosurgeons, and emergency medicine doctors	97.91	98.76	NA	NA	Detection/per-scan
Choi 2024 [39]	Retrospective	ICH	Local dataset/tested on 2146 NCCTs	DL	Board-certified neuroradiologists	59.2	91.6	83.3	NA	Detection/per-scan
Colasurdo 2022 [40]	Retrospective	SDH	Local dataset/tested on 263 NCCTs	Deep-CNN	Experienced neuroradiologists	91.4	96.4	95.1	NA	Detection/per-scan
Coorens 2023 [41]	Retrospective	ICH	Local datasets/trained and validated on 16,348 NCCTs/tested on 4095 NCCTs	Masked loss U-Net architecture	NA	77	96.2	91.1	NA	Detection/per-slice
Cortés-Ferre 2023 [42]	Retrospective	ICH	Trained on the RSNA dataset/55 NCCTs were used for external validation	DL	Experienced radiologists, neurologists and neurocritical doctors	100	100	100	NA	Detection/per-scan
D’Angelo 2024 [28]	Retrospective	ICH	Local dataset/502 NCCTs	Dense-UNet architecture	Board-certified radiologists	90.37	94.85	91.24	NA	Detection/per-scan
Dawud 2019 [43]	Retrospective	ICH	Local dataset/Trained on 8855 and tested on 3790 NCCTs	AlexNet-SVM	NA	95	90	93	NA	Detection/per-slice
Del Gaizo 2024 [44]	Retrospective	ICH	Local dataset/58,321 NCCTs	CINA v1.0 device (DL)	Experienced emergency radiologists	75.6	92.1	91.7	NA	Detection/per-scan
Dyer 2021 [45]	Retrospective	ICH	Tested on a local dataset consisting of 390 NCCTs	EfficientNet neural network architecture	Consultant radiologists and neuroradiologists	98.8	92.5	NA	98.8	Detection/per-scan
Eldaya 2022 [46]	Retrospective	ICH	Tested on a local dataset consisting of 307 NCCTs	RAPID AI software (hybrid deep 2D–3D DCNN)	Board-certified or board-eligible neuroradiologists	91.9	84.4	85.3	NA	Detection/per-scan
Gibson 2022 [47]	Retrospective	ICH	Trained on 25,946 NCCTs/evaluated on 16,764 NCCTs from RSNA ICH dataset	Deep neural networks	Neuroradiologists	86	92	NA	0.95	Detection/per-scan
Ginat 2019 [48]	Prospective	ICH	Local dataset/tested on 2011 NCCTs	Aidoc (CNN)	Board-certified neuroradiologist	88.7	94.2	93.4	NA	Detection/per-scan
Ginat 2021 [49]	Prospective	ICH	Local dataset/tested on 8723 NCCTs	Aidoc (CNN)	Radiologist report	88.4	96.1	NA	NA	Detection/per-scan
Gruschwitz 2021 [50]	Retrospective/ prospective	ICH	Retrospective (Local dataset/tested on 872 NCCTs) Prospective (Local dataset/tested on 100 NCCTs)	Dense-UNet architecture	A resident radiologist with subsequent supervision of a board-certified radiologist				NA	Detection/per-scan Retrospective (sensitivity = 91.4/specificity = 90.4/accuracy = 90.8) Prospective (sensitivity = 81.8/specificity = 97.8/accuracy = 96)
Guo 2022 [51]	Prospective	ICH	Trained on a local dataset/tested on a local dataset consisting of 650 NCCTs	RoLo, a novel weakly supervised deep learning algorithm	Expert radiologists	97.9	91.5	NA	0.987	Detection/per-scan
He 2024 [52]	Retrospective	ICH	Trained and tested on the ASNR public dataset consisting of 750,000 head CTs	End-to-end deep multiscale convolutional feature fusion framework	Radiologists	NA	NA	NA	0.988	Detection/per-slice
Heit 2021 [53]	Retrospective	ICH	Trained on a local dataset including 805 NCCTs/validated on a local dataset consisting of 308 NCCTs	Hybrid deep 2D–3D CNN	Expert neuroradiologists	95.6	95.3	NA	NA	Detection/per-scan/performance on validation test is reported
Hofmeijer 2023 [54]	Retrospective	ICH	Trained and tested on a local dataset including 134 NCCTs	CNN	Neuroradiologists	NA	NA	NA	0.82	Detection/per-scan
Hopkins 2022 [55]	Prospective	ICH	Trained on RSNA dataset containing 21,784 NCCTs/tested on a local dataset including 726 NCCTs	CNN	Board-certified radiologists	94	94	NA	0.98	Detection/per-scan
Hu 2023 [7]	Ambispective	ICH	Three local datasets for external validation/trained on 931 NCCTs	Hybrid 2D/3D UNet deep-learning framework	Neurosurgeons and radiologists (not well defined)	81.2	99.9	99.7	NA	Detection/per-scan
Kang 2023 [56]	Retrospective	ICH	Trained on two publicly available datasets (RSNA and AI-Hub)/two local datasets including 986 NCCTs were used for external testing	2D U-net with the Inception module + weighted ensemble	Vascular neurologist and neuroradiologists	92.8	85.7	NA	95.3	Detection/per-scan
Kau 2021 [57]	Retrospective	ICH	Local dataset/tested on 2188 NCCTs	Aidoc (CNN)	Board-certified neuroradiologist	68.2	96.8	94	NA	Detection/per-scan
Kiefer 2024 [58]	Retrospective	ICH	Tested on a local dataset including 432 NCCTs	Dense-UNet architecture	Experienced radiologists	98.1	89.7	90.7	NA	Detection/per-scan
Kumaravel 2020 [26]	Retrospective	ICH	Trained and tested on a publicly available dataset (CQ500 with 451 NCCTs)	DCNN (AlexNet-SVM)	Experienced radiologists	99.86	99.86	99.86	0.999	Detection/per-slice
Kundisch 2021 [59]	Retrospective	ICH	Tested on a local dataset including 4946 NCCTs	3D DCNN (Aidoc)	Neuroradiologists	NA	NA	NA	NA	Detection/per-scan
Kuo 2019 [60]	Retrospective	ICH	Local datasets/Trained on 4396 head CTs/tested on 200 head CTs	Patch-based fully convolutional neural network (PatchFCN)	Board-certified neuroradiologist	100	90	NA	0.991 ± 0.006	Detection/per-scan
Lee 2018 [61]	Retrospective	ICH	Local datasets/Trained on 904 NCCTs/tested on 200 head CTs	DCNNs	Board-certified neuroradiologists	98	95	NA	99.3	Detection and classification/per-scan
Lee 2020 [62]	Retrospective	ICH	Local datasets/trained on 166 NCCTs/validation set included 84 cases	ANN	Board-certified neuroradiologists	78	80	NA	0.859	Detection/per-scan/performance on validation dataset is reported
López-Pérez 2022 [63]	Retrospective	ICH	Trained and tested on RSNA dataset	Multiple Instance Learning based on Deep Gaussian Processes (DGPMIL)	Neuroradiologists	95.7	NA	82.5	NA	Detection/per-scan
Majumdar 2018 [64]	Retrospective	ICH	Local dataset/trained on 60 NCCTs with ICH/tested on 69 NCCTs	CNN (modified U-Net)	Experienced radiologists	81	98	NA	NA	Sensitivity per lesion and specificity per normal case
McLouth 2021 [65]	Retrospective	ICH	Local dataset/tested on 814 NCCTs	CINA v1.0 device (DL)	Board-certified neuroradiologists	91.4	97.5	95.6	NA	Detection/per-scan True positive rate (%) for ICHs < 5, 5–25, and >25 mL is 71.8, 100, and 100, respectively
Monteiro 2020 [66]	Retrospective	ICH	Trained and tested on the CENTERTBI dataset (184 NCCTs in the training subset and 655 scans in the test subset)/external validation on a publicly available dataset (CQ500 dataset)	CNN	Experienced radiologists	90	51	NA	0.83	Detection/per-scan/performance on CQ500 dataset is reported
Nada 2024 [67]	Prospective	ICH	Local dataset/tested on 5600 NCCTs	Aidoc (CNN)	Experienced neuroradiologists	89	96	94	0.954	Detection/per-scan
Neves 2023 [27]	Retrospective	ICH	Local dataset/tested on 510 NCCTs	Caire ICH vR1 (Caire Health Inc.)	Experienced radiologists	97.52	100	98.05	0.9957	Detection/per-scan
Nishi 2021 [68]	Retrospective	Non-traumatic SAH	Local datasets/trained on 757 NCCTs/tested on 331 NCCTs	3D U-net (CNN)	Neurosurgery specialists	99	92	95	0.99	Detection/per-scan
Pettet 2024 [69]	Retrospective	ICH	Local dataset consisting of 1315 NCCTs	CNN (qER, developed by Qure.ai)	Experienced neuroradiologists and general radiologists	85.7	94.3	93.5	NA	Detection/per-scan
Phaphuangwittayakul 2022 [70]	Retrospective	ICH	Two public datasets (RSNA and PhysioNet) and one private dataset (CMU-TBI) including 321 NCCTs	CNN	NA	95.77	96.9	96.21	NA	Detection/per-slice
Rao 2022 [71]	Retrospective	ICH	Local dataset including 1164 NCCT images/Trained on 931 NCCT images/tested on 233 NCCT images	ResNet-based transfer learning model	Radiologists	99.4	99.7	99.6	100	Detection/per-slice
Roshan 2024 [72]	Retrospective	ICH	Local dataset including 4203 NCCTs	CNN (Viz.ai ICH)	Experienced neuroradiologists	85.3	98.3	NA	NA	Detection/per-scan Viz.ai ICH for intraparenchymal ICH >5 mL (99%) showed higher sensitivity than ICH <5 mL (99% and 84%, respectively). For SDH >10 mL it exhibited higher sensitivity than ICH <10 mL (90% and 77%, respectively)
Salehinejad 2021 [73]	Retrospective	ICH	Trained on 21,784 NCCTs from the RSNA dataset/external validated on a dataset including 5965 NCCTs	DCNN	A trained research assistant	91.3	94.1	93.8	0.954	Detection/per-scan/performance on external validation dataset is reported
Savage 2024 [74]	Prospective	ICH	Tested on a local dataset consisting of 3716 NCCTs	Aidoc (CNN)	Attending radiologists	87.8	94.3	93	NA	Detection/per-scan
Schmitt 2021 [75]	Retrospective	ICH	Local dataset/tested on 160 NCCTs	Brainomix algorithm (DCNN)	Board-certified neuroradiologist	91	89	NA	0.9	Detection/per-scan
Seyam 2022 [76]	Prospective	ICH	Local dataset/tested on 3017 NCCTs	Aidoc (CNN)	Board-certified neuroradiologist	87.2	93.9	93	NA	Detection/per-scan
Sindhura 2023 [77]	Retrospective	ICH	RSNA dataset (6122 NCCTs were used for training, 765 NCCTs were used for validation, and 765 NCCTs were used for testing)	Joint CNN-RNN	Neuroradiologists	93.16	97.1	95.5	NA	Detection/patient-level
Sreekrishnan 2023 [78]	Retrospective	ICH	Tested on a local dataset including 881 NCCTs	RAPID AI software (hybrid 2D–3D DCNN)	Neuroradiologists	97.84	99.52	NA	NA	Detection/per-scan
Teneggi 2024 [79]	Retrospective	ICH	Trained on 17,388 NCCTs/tested on two local datasets (CQ500 and CT-ICH with 436 and 75 NCCTs, respectively)	Attention-based CNN	Expert radiologists	NA	NA	NA	NA	Detection/per-scan/for CQ500 datset AUC = 0.92 and for CT-ICH dataset AUC = 0.95)
Thanellas 2023 [80]	Retrospective	SAH	Local datasets/trained on 1083 NCCTs/tested on two public external validation datasets (Zurich and CQ500 datasets) including 1379 NCCTs	CNN (U-Net)	NA for Zurich dataset and expert radiologists for CQ500 dataset	99	63	67	NA	Detection/per-scan
Tharek 2022 [81]	Retrospective	ICH	Trained and tested on a public dataset including 200 NCCTs (100 with ICH and 100 without)	CNN	NA	96.94	93.14	95	NA	Detection/per-scan
Trang 2024 [82]	Retrospective	ICH	Tested on a local datset consisting of 9736 NCCTs	Aidoc (CNN)	Board-certified or board-eligible neuroradiologists	85.2	94.3	93.1	NA	Detection/per-scan
Villringer 2024 [83]	Retrospective	ICH	Trained on 674,000 NCCT slices from RSNA/tested on 255 NCCTs from a local dataset	CNN + Efficient-Net-B3 architecture	Experienced radiologists	90	96	96	NA	Detection/per-scan/performance is the mean performance of AI vs. expert raters
Voter 2021 [84]	Retrospective	ICH	Tested on a local dataset including 3605 NCCTs	Aidoc (CNN)	CAQ-certified neuroradiologist	92.3	97.7	NA	NA	Detection/per-scan
Wang 2021 [85]	Retrospective	ICH	Trained on RSNA dataset/tested on two publicly available external datasets (PhysioNet-ICH with 75 and CQ500 with 491 NCCTs)	2D CNN	Experienced radiologists			NA		Detection/per-scan Performance on PhysioNet-ICH (sensitivity = 88.7/specificity = 94.4/AUC = 0.964) Performance on CQ500 dataset is reported (sensitivity = 91.4/specificity = 84.4/AUC = 0.949)
Wang 2023 [86]	Retrospective	ICH	Trained on 20,000 NCCTs from different centers/tested on a local dataset consisting of 527 NCCTs	VeriScout™ tool (CNN)	Radiology trainee with 2 years of specialty experience + a sub-specialty neuroradiologist	92	96	96	NA	Detection/per-scan
Wang 2024 [87]	Retrospective	ICH	Trained and tested on local datasets/validated on a local dataset including 778 NCCTs	2D CNN with modifications from U-Net and ResNet architectures	Two radiologists and one neurosurgeon	97.7	98.71	98.2	NA	Detection/per-scan/performance of model on validation dataset is reported
Wu 2024 [88]	Retrospective	ICH	Training + Cross-Validation on a local dataset including 10,699 NCCTs/CQ500 dataset including 491 NCCTs used for external testing	Transfer learning with 2D CNN + bidirectional LSTM + attention layer at study level	experienced radiologists	88.5	89.6	91.6	0.96	Detection/per-scan/performance of model on external dataset (CQ500) is reported
Ye 2019 [89]	Retrospective	ICH	Trained on a local dataset containing 2836 NCCTs/tested on 299 NCCTs	3D Joint CNN-RNN	senior radiologists	98	99	99	1	Detection/per-scan
Yedavalli 2023 [90]	Retrospective	ICH	Tested on a local dataset including 243 NCCTs	RAPID AI software (hybrid 2D–3D CNN)	board-certified general and neuroradiologists	96.2	99.5	NA	NA	Detection/per-scan
Yeo 2023 [91]	Retrospective	ICH	Trained on the public Kaggle dataset and tested on the CQ500 dataset with 491 NCCTs	Joint CNN-RNN	Experienced radiologists	95	81	88	0.966	Detection/per-scan
Yun 2023 [92]	Retrospective	ICH	Trained and validated on a local dataset including 104,666 NCCT slices/external validated on a local dataset consisting of 49,841 NCCTs	Joint CNN-RNN	Neuroradiologists	94.4	98.2	97.7	0.992	Detection/per-scan
Zhou 2022 [93]	Retrospective	ICH	Local dataset/351 NCCTs	DL (Res-Net18 and Dense-Net121)	Experienced radiologists			NA	NA	Detection/per-slice ResNet-18 (sensitivity of 100% and specificity of 87%) DenseNet-121 (sensitivity of 98% and specificity of 79%)
Zia 2022 [94]	Retrospective/ prospective	ICH	Tested on a local dataset including 1446 NCCTs	Aidoc (CNN)	Board-certified neuroradiologist	85.7	96.8	NA	NA	Detection/per-scan/performance of prospective dataset is reported

ICH; Intracranial hemorrhage, SDH; Subdural hematoma, SAH; Subarachnoid hemorrhage, DL; Deep learning, CNN; Convolutional neural networks, RNN; Recurrent neural networks, LSTM; Long Short-Term Memory, FCN; Fully convolutional networks, DCNN; Deep convolutional neural networks, AUC; Area under curve, PPV; Positive predictive value, NA; Not available.

## Data Availability

No new data were created in this study. All required data are available in the included studies.

## Supplementary Materials

The following supporting information can be downloaded at: https://www.mdpi.com/article/10.3390/jcm14072377/s1, Table S1: Quality Assessment of Diagnostic Accuracy Studies-2 (QUADS-2) risk of bias assessment. Figure S1: Funnel plots of sensitivity (a), specificity (b), PPV (c), and NPV (d).

## Abbreviations

The following abbreviations are used in this manuscript:

ICH	Intracranial hemorrhage
AI	Artificial intelligence
DL	Deep learning
NCCT	Non-contrast computed tomography
ANN	Artificial neural networks
CNN	Convolutional neural network
IPH	Intraparenchymal hemorrhage
SDH	Subdural hemorrhage
EDH	Epidural hemorrhage
IVH	Intraventricular hemorrhage
SAH	Subarachnoid hemorrhage
AUC	Area under curve
TP	True positive
TN	True negative
FP	False positive
FN	False negative
PPV	Positive predictive value
NPV	Negative predictive value
RNN	Recurrent neural networks
LSTM	Long Short-Term Memory
FCN	Fully convolutional networks
DCNN	Deep convolutional neural networks

## References

[1] Caceres J.A., Goldstein J.N. (2012). Intracranial hemorrhage. Emerg. Med. Clin. N. Am..

[2] Fernando S.M., Qureshi D., Talarico R., Tanuseputro P., Dowlatshahi D., Sood M.M., Smith E.E., Hill M.D., McCredie V.A., Scales D.C. (2021). Intracerebral Hemorrhage Incidence, Mortality, and Association with Oral Anticoagulation Use. Stroke.

[3] Woo D., Comeau M.E., Venema S.U., Anderson C.D., Flaherty M., Testai F., Kittner S., Frankel M., James M.L., Sung G. (2022). Risk Factors Associated with Mortality and Neurologic Disability After Intracerebral Hemorrhage in a Racially and Ethnically Diverse Cohort. JAMA Netw. Open.

[4] Goldstein J.N., Gilson A.J. (2011). Critical care management of acute intracerebral hemorrhage. Curr. Treat. Options Neurol..

[5] Alobeidi F., Aviv R.I. (2015). Emergency Imaging of Intracerebral Haemorrhage. Front. Neurol. Neurosci..

[6] Rajashekar D., Liang J.W. (2025). Intracerebral Hemorrhage. StatPearls [Internet].

[7] Hu P., Zhou H., Yan T., Miu H., Xiao F., Zhu X., Shu L., Yang S., Jin R., Dou W. (2023). Deep learning-assisted identification and quantification of aneurysmal subarachnoid hemorrhage in non-contrast CT scans: Development and external validation of Hybrid 2D/3D UNet. Neuroimage.

[8] Frija G., Blažić I., Frush D.P., Hierath M., Kawooya M., Donoso-Bach L., Brkljačić B. (2021). How to improve access to medical imaging in low- and middle-income countries?. Eclinicalmedicine.

[9] Sage A., Badura P. (2020). Intracranial Hemorrhage Detection in Head CT Using Double-Branch Convolutional Neural Network, Support Vector Machine, and Random Forest. Appl. Sci..

[10] Kwee T.C., Kwee R.M. (2021). Workload of diagnostic radiologists in the foreseeable future based on recent scientific advances: Growth expectations and role of artificial intelligence. Insights Into Imaging.

[11] Strub W., Leach J., Tomsick T., Vagal A. (2007). Overnight preliminary head CT interpretations provided by residents: Locations of misidentified intracranial hemorrhage. AJNR Am. J. Neuroradiol..

[12] Erly W.K., Berger W.G., Krupinski E., Seeger J.F., Guisto J.A. (2002). Radiology resident evaluation of head CT scan orders in the emergency department. AJNR Am. J. Neuroradiol..

[13] Arendts G., Manovel A., Chai A. (2003). Cranial CT interpretation by senior emergency department staff. Australas. Radiol..

[14] Amisha Malik P., Pathania M., Rathaur V.K. (2019). Overview of artificial intelligence in medicine. J. Family Med. Prim. Care.

[15] Al Kuwaiti A., Nazer K., Al-Reedy A., Al-Shehri S., Al-Muhanna A., Subbarayalu A.V., Al Muhanna D., Al-Muhanna F.A. (2023). A Review of the Role of Artificial Intelligence in Healthcare. J. Pers. Med..

[16] Sarker I.H. (2021). Deep Learning: A Comprehensive Overview on Techniques, Taxonomy, Applications and Research Directions. SN Comput. Sci..

[17] LeCun Y., Bengio Y., Hinton G. (2015). Deep learning. Nature.

[18] Cao H., Morotti A., Mazzacane F., Desser D., Schlunk F., Güttler C., Kniep H., Penzkofer T., Fiehler J., Hanning U. (2023). External Validation and Retraining of DeepBleed: The First Open-Source 3D Deep Learning Network for the Segmentation of Spontaneous Intracerebral and Intraventricular Hemorrhage. J. Clin. Med..

[19] Angkurawaranon S., Sanorsieng N., Unsrisong K., Inkeaw P., Sripan P., Khumrin P., Angkurawaranon C., Vaniyapong T., Chitapanarux I. (2023). A comparison of performance between a deep learning model with residents for localization and classification of intracranial hemorrhage. Sci. Rep..

[20] Alis D., Alis C., Yergin M., Topel C., Asmakutlu O., Bagcilar O., Senli Y.D., Ustundag A., Salt V., Dogan S.N. (2022). A joint convolutional-recurrent neural network with an attention mechanism for detecting intracranial hemorrhage on noncontrast head CT. Sci. Rep..

[21] Mazurowski M.A., Buda M., Saha A., Bashir M.R. (2019). Deep learning in radiology: An overview of the concepts and a survey of the state of the art with focus on MRI. J. Magn. Reson. Imaging.

[22] Sarvamangala D.R., Kulkarni R.V. (2022). Convolutional neural networks in medical image understanding: A survey. Evol. Intell..

[23] Kugunavar S., Prabhakar C.J. (2021). Convolutional neural networks for the diagnosis and prognosis of the coronavirus disease pandemic. Vis. Comput. Ind. Biomed. Art.

[24] Altuve M., Pérez A. (2022). Intracerebral hemorrhage detection on computed tomography images using a residual neural network. Phys Med..

[25] Cho J., Park K.-S., Karki M., Lee E., Ko S., Kim J.K., Lee D., Choe J., Son J., Kim M. (2019). Improving Sensitivity on Identification and Delineation of Intracranial Hemorrhage Lesion Using Cascaded Deep Learning Models. J. Digit. Imaging.

[26] Kumaravel P., Mohan S., Arivudaiyanambi J., Shajil N., Venkatakrishnan H.N. (2021). A Simplified Framework for the Detection of Intracranial Hemorrhage in CT Brain Images Using Deep Learning. Curr. Med. Imaging.

[27] Neves G., Warman P.I., Warman A., Warman R., Bueso T., Vadhan J.D., Windisch T. (2023). External Validation of an Artificial Intelligence Device for Intracranial Hemorrhage Detection. World Neurosurg..

[28] D’angelo T., Bucolo G.M., Kamareddine T., Yel I., Koch V., Gruenewald L.D., Martin S., Alizadeh L.S., Mazziotti S., Blandino A. (2024). Accuracy and time efficiency of a novel deep learning algorithm for Intracranial Hemorrhage detection in CT Scans. Radiol. Med..

[29] Chien H.C., Yang T.L., Juang W.C., Chen Y.A., Li Y.J., Chen C.Y. (2022). Pilot Report for Intracranial Hemorrhage Detection with Deep Learning Implanted Head Computed Tomography Images at Emergency Department. J. Med. Syst..

[30] Whiting P.F., Rutjes A.W.S., Westwood M.E., Mallett S., Deeks J.J., Reitsma J.B., Leeflang M.M.G., Sterne J.A.C., Bossuyt P.M.M. (2011). QUADAS-2: A revised tool for the quality assessment of diagnostic accuracy studies. Ann. Intern. Med..

[31] Melsen W.G., Bootsma M.C., Rovers M.M., Bonten M.J. (2014). The effects of clinical and statistical heterogeneity on the predictive values of results from meta-analyses. Clin. Microbiol. Infect..

[32] Abrigo J.M., Ko K.-L., Chen Q., Lai B.M., Cheung T.C., Chu W.C., Yu S.C. (2023). Artificial intelligence for detection of intracranial haemorrhage on head computed tomography scans: Diagnostic accuracy in Hong Kong. Hong. Kong Med. J..

[33] Arbabshirani M.R., Fornwalt B.K., Mongelluzzo G.J., Suever J.D., Geise B.D., Patel A.A., Moore G.J. (2018). Advanced machine learning in action: Identification of intracranial hemorrhage on computed tomography scans of the head with clinical workflow integration. Npj Digit. Medicine.

[34] Arman S.E., Rahman S.S., Irtisam N., Deowan S.A., Islam A., Sakib S., Hasan M. (2023). Intracranial Hemorrhage Classification From CT Scan Using Deep Learning and Bayesian Optimization. IEEE Access.

[35] Babu P.P.S., Brindha T. (2024). Deep Learning Fusion for Intracranial Hemorrhage Classification in Brain CT Imaging. Int. J. Adv. Comput. Sci. Appl. (IJACSA).

[36] Buls N., Watté N., Nieboer K., Ilsen B., de Mey J. (2021). Performance of an artificial intelligence tool with real-time clinical workflow integration—Detection of intracranial hemorrhage and pulmonary embolism. Phys. Med..

[37] Chang P., Kuoy E., Grinband J., Weinberg B., Thompson M., Homo R., Chen J., Abcede H., Shafie M., Sugrue L. (2018). Hybrid 3D/2D Convolutional Neural Network for Hemorrhage Evaluation on Head CT. AJNR Am. J. Neuroradiol..

[38] Chilamkurthy S., Ghosh R., Tanamala S., Biviji M., Campeau N.G., Venugopal V.K., Mahajan V., Rao P., Warier P. (2018). Deep learning algorithms for detection of critical findings in head CT scans: A retrospective study. Lancet.

[39] Choi S.Y., Kim J.H., Chung H.S., Lim S., Kim E.H., Choi A. (2024). Impact of a deep learning-based brain CT interpretation algorithm on clinical decision-making for intracranial hemorrhage in the emergency department. Sci. Rep..

[40] Colasurdo M., Leibushor N., Robledo A., Vasandani V., Luna Z.A., Rao A.S., Garcia R., Srinivasan V.M., Sheth S.A., Avni N. (2023). Automated detection and analysis of subdural hematomas using a machine learning algorithm. J. Neurosurg..

[41] Coorens N.A.M., Lipman K.G.M., Krishnam S.P.M., Tan C.O., Alic L., Gupta R. (2023). Intracerebral Hemorrhage Segmentation on Noncontrast Computed Tomography Using a Masked Loss Function U-Net Approach. J. Comput. Assist. Tomogr..

[42] Cortés-Ferre L., Gutiérrez-Naranjo M.A., Egea-Guerrero J.J., Pérez-Sánchez S., Balcerzyk M. (2023). Deep Learning Applied to Intracranial Hemorrhage Detection. J. Imaging.

[43] Dawud A.M., Yurtkan K., Oztoprak H. (2019). Application of Deep Learning in Neuroradiology: Brain Haemorrhage Classification Using Transfer Learning. Comput. Intell. Neurosci..

[44] Del Gaizo A.J., Osborne T.F., Shahoumian T., Sherrier R. (2024). Deep Learning to Detect Intracranial Hemorrhage in a National Teleradiology Program and the Impact on Interpretation Time. Radiol Artif Intell..

[45] Dyer T., Chawda S., Alkilani R., Morgan T.N., Hughes M., Rasalingham S. (2022). Validation of an artificial intelligence solution for acute triage and rule-out normal of non-contrast CT head scans. Neuroradiology.

[46] Eldaya R.W.M., Kansagra A.P., Zei M., Mason E.D., Holder D., Heitsch L., Vo K.D., Goyal M.S.M. (2022). Performance of Automated RAPID Intracranial Hemorrhage Detection in Real-World Practice: A Single-Institution Experience. J. Comput. Assist. Tomogr..

[47] Gibson E., Georgescu B., Ceccaldi P., Trigan P.-H., Yoo Y., Das J., Re T.J., Rs V., Balachandran A., Eibenberger E. (2022). Artificial Intelligence with Statistical Confidence Scores for Detection of Acute or Subacute Hemorrhage on Noncontrast CT Head Scans. Radiol. Artif. Intell..

[48] Ginat D.T. (2020). Analysis of head CT scans flagged by deep learning software for acute intracranial hemorrhage. Neuroradiology..

[49] Ginat D. (2021). Implementation of Machine Learning Software on the Radiology Worklist Decreases Scan View Delay for the Detection of Intracranial Hemorrhage on CT. Brain Sci..

[50] Gruschwitz P., Grunz J.-P., Kuhl P.J., Kosmala A., Bley T.A., Petritsch B., Heidenreich J.F. (2021). Performance testing of a novel deep learning algorithm for the detection of intracranial hemorrhage and first trial under clinical conditions. Neurosci. Inform..

[51] Guo Y., Guo Y., He Y., He Y., Lyu J., Lyu J., Zhou Z., Zhou Z., Yang D., Yang D. (2022). Deep learning with weak annotation from diagnosis reports for detection of multiple head disorders: A prospective, multicentre study. Lancet Digit. Health.

[52] He B., Xu Z., Zhou D., Zhang L. (2024). Deep multiscale convolutional feature learning for intracranial hemorrhage classification and weakly supervised localization. Heliyon.

[53] Heit J., Coelho H., Lima F., Granja M., Aghaebrahim A., Hanel R., Kwok K., Haerian H., Cereda C., Venkatasubramanian C. (2021). Automated Cerebral Hemorrhage Detection Using RAPID. AJNR Am. J. Neuroradiol..

[54] Hofmeijer E., Tan C., van der Heijden F., Gupta R. (2023). Crowd-Sourced Deep Learning for Intracranial Hemorrhage Identification: Wisdom of Crowds or Laissez-Faire. AJNR Am. J. Neuroradiol..

[55] Hopkins B.S., Murthy N.K., Texakalidis P., Karras C.L., Mansell M., Jahromi B.S., Potts M.B., Dahdaleh N.S. (2022). Mass Deployment of Deep Neural Network: Real-Time Proof of Concept with Screening of Intracranial Hemorrhage Using an Open Data Set. Neurosurgery.

[56] Kang D.-W., Park G.-H., Ryu W.-S., Schellingerhout D., Kim M., Kim Y.S., Park C.-Y., Lee K.-J., Han M.-K., Jeong H.-G. (2023). Strengthening deep-learning models for intracranial hemorrhage detection: Strongly annotated computed tomography images and model ensembles. Front. Neurol..

[57] Kau T., Ziurlys M., Taschwer M., Kloss-Brandstätter A., Grabner G., Deutschmann H. (2022). FDA-approved deep learning software application versus radiologists with different levels of expertise: Detection of intracranial hemorrhage in a retrospective single-center study. Neuroradiology.

[58] Kiefer J., Kopp M., Ruettinger T., Heiss R., Wuest W., Amarteifio P., Stroebel A., Uder M., May M.S. (2023). Diagnostic Accuracy and Performance Analysis of a Scanner-Integrated Artificial Intelligence Model for the Detection of Intracranial Hemorrhages in a Traumatology Emergency Department. Bioengineering.

[59] Kundisch A., Hönning A., Mutze S., Kreissl L., Spohn F., Lemcke J., Sitz M., Sparenberg P., Goelz L. (2021). Deep learning algorithm in detecting intracranial hemorrhages on emergency computed tomographies. PLoS ONE.

[60] Kuo W., Häne C., Mukherjee P., Malik J., Yuh E.L. (2019). Expert-level detection of acute intracranial hemorrhage on head computed tomography using deep learning. Proc. Natl. Acad. Sci. USA.

[61] Lee H., Yune S., Mansouri M., Kim M., Tajmir S.H., Guerrier C.E., Ebert S.A., Pomerantz S.R., Romero J.M., Kamalian S. (2019). An explainable deep-learning algorithm for the detection of acute intracranial haemorrhage from small datasets. Nat. Biomed. Eng..

[62] Lee J.Y., Kim J.S., Kim T.Y., Kim Y.S. (2020). Detection and classification of intracranial haemorrhage on CT images using a novel deep-learning algorithm. Sci. Rep..

[63] López-Pérez M., Schmidt A., Wu Y., Molina R., Katsaggelos A.K. (2022). Deep Gaussian processes for multiple instance learning: Application to CT intracranial hemorrhage detection. Comput. Methods Programs Biomed.

[64] Majumdar A., Brattain L., Telfer B., Farris C., Scalera J. Detecting Intracranial Hemorrhage with Deep Learning. Proceedings of the 2018 40th Annual International Conference of the IEEE Engineering in Medicine and Biology Society (EMBC).

[65] McLouth J., Elstrott S., Chaibi Y., Quenet S., Chang P.D., Chow D.S., Soun J.E. (2021). Validation of a Deep Learning Tool in the Detection of Intracranial Hemorrhage and Large Vessel Occlusion. Front. Neurol..

[66] Monteiro M., Newcombe V.F.J., Mathieu F., Adatia K., Kamnitsas K., Ferrante E., Das T., Whitehouse D., Rueckert D., Menon D.K. (2020). Multiclass semantic segmentation and quantification of traumatic brain injury lesions on head CT using deep learning: An algorithm development and multicentre validation study. Lancet Digit. Health.

[67] Nada A., Sayed A.A., Hamouda M., Tantawi M., Khan A., Alt A., Hassanein H., Sevim B.C., Altes T., Gaballah A. (2024). External validation and performance analysis of a deep learning-based model for the detection of intracranial hemorrhage. Neuroradiol. J..

[68] Nishi T., Yamashiro S., Okumura S., Takei M., Tachibana A., Akahori S., Kaji M., Uekawa K., Amadatsu T. (2021). Artificial Intelligence Trained by Deep Learning Can Improve Computed Tomography Diagnosis of Nontraumatic Subarachnoid Hemorrhage by Nonspecialists. Neurol. Med. Chir..

[69] Pettet G., West J., Robert D., Khetani A., Kumar S., Golla S., Lavis R. (2024). A retrospective audit of an artificial intelligence software for the detection of intracranial haemorrhage used by a teleradiology company in the United Kingdom. BJR Open..

[70] Phaphuangwittayakul A., Guo Y., Ying F., Dawod A.Y., Angkurawaranon S., Angkurawaranon C. (2022). An optimal deep learning framework for multi-type hemorrhagic lesions detection and quantification in head CT images for traumatic brain injury. Appl. Intell..

[71] Rao B.N., Mohanty S., Sen K., Acharya U.R., Cheong K.H., Sabut S. (2022). Deep Transfer Learning for Automatic Prediction of Hemorrhagic Stroke on CT Images. Comput. Math. Methods Med..

[72] Roshan M.P., A Al-Shaikhli S., Linfante I., Antony T.T., E Clarke J., Noman R., Lamy C., Britton S., Belnap S.C., Abrams K. (2024). Revolutionizing Intracranial Hemorrhage Diagnosis: A Retrospective Analytical Study of Viz.ai ICH for Enhanced Diagnostic Accuracy. Cureus.

[73] Salehinejad H., Kitamura J., Ditkofsky N., Lin A., Bharatha A., Suthiphosuwan S., Lin H.-M., Wilson J.R., Mamdani M., Colak E. (2021). A real-world demonstration of machine learning generalizability in the detection of intracranial hemorrhage on head computerized tomography. Sci. Rep..

[74] Savage C.H., Tanwar M., Elkassem A.A., Sturdivant A., Hamki O., Sotoudeh H., Sirineni G., Singhal A., Milner D., Jones J. (2024). Prospective Evaluation of Artificial Intelligence Triage of Intracranial Hemorrhage on Noncontrast Head CT Examinations. AJR Am. J. Roentgenol..

[75] Schmitt N., Mokli Y., Weyland C.S., Gerry S., Herweh C., Ringleb P.A., Nagel S. (2022). Automated detection and segmentation of intracranial hemorrhage suspect hyperdensities in non-contrast-enhanced CT scans of acute stroke patients. Eur. Radiol..

[76] Seyam M., Weikert T., Sauter A., Brehm A., Psychogios M.-N., Blackham K.A. (2022). Utilization of Artificial Intelligence-based Intracranial Hemorrhage Detection on Emergent Noncontrast CT Images in Clinical Workflow. Radiol. Artif. Intell..

[77] Sindhura C., Al Fahim M., Yalavarthy P.K., Gorthi S. (2024). Fully automated sinogram-based deep learning model for detection and classification of intracranial hemorrhage. Med. Phys..

[78] Sreekrishnan A., Giurgiutiu D.-V., Kitamura F., Martinelli C., Abdala N., Haerian H., Dehkharghani S., Kwok K., Yedavalli V., Heit J.J. (2023). Decreasing false-positive detection of intracranial hemorrhage (ICH) using RAPID ICH 3. J. Stroke Cerebrovasc. Dis..

[79] Teneggi J., Yi P.H., Sulam J. (2024). Examination-Level Supervision for Deep Learning-based Intracranial Hemorrhage Detection on Head CT Scans. Radiol. Artif. Intell..

[80] Thanellas A., Peura H., Lavinto M., Ruokola T., Vieli M., Staartjes V.E., Winklhofer S., Serra C., Regli L., Korja M. (2023). Development and External Validation of a Deep Learning Algorithm to Identify and Localize Subarachnoid Hemorrhage on CT Scans. Neurology.

[81] Tharek A., Muda A.S., Hudi A.B., Hudin A.B. (2022). Intracranial Hemorrhage Detection in Ct Scan Using Deep Learning. Asian J. Med. Technol..

[82] Trang A., Putman K., Savani D., Chatterjee D., Zhao J., Kamel P., Jeudy J.J., Parekh V.S., Yi P.H. (2024). Sociodemographic biases in a commercial AI model for intracranial hemorrhage detection. Emerg. Radiol..

[83] Villringer K., Sokiranski R., Opfer R., Spies L., Hamann M., Bormann A., Brehmer M., Galinovic I., Fiebach J.B. (2025). An Artificial Intelligence Algorithm Integrated into the Clinical Workflow Can Ensure High Quality Acute Intracranial Hemorrhage CT Diagnostic. Clin. Neuroradiol..

[84] Voter A.F., Meram E., Garrett J.W., Yu J.-P.J. (2021). Diagnostic Accuracy and Failure Mode Analysis of a Deep Learning Algorithm for the Detection of Intracranial Hemorrhage. J. Am. Coll. Radiol..

[85] Wang X., Shen T., Yang S., Lan J., Xu Y., Wang M., Zhang J., Han X. (2021). A deep learning algorithm for automatic detection and classification of acute intracranial hemorrhages in head CT scans. NeuroImage Clin..

[86] Wang D., Jin R., Shieh C.-C., Ng A.Y., Pham H., Dugal T., Barnett M., Winoto L., Wang C., Barnett Y. (2023). Real world validation of an AI-based CT hemorrhage detection tool. Front. Neurol..

[87] Wang H.-C., Wang S.-C., Xiao F., Ho U.-C., Lee C.-H., Yan J.-L., Chen Y.-F., Ko L.-W. (2025). Development of a Clinically Applicable Deep Learning System Based on Sparse Training Data to Accurately Detect Acute Intracranial Hemorrhage from Non-enhanced Head Computed Tomography. Neurol. Med. Chir..

[88] Wu Y., Iorga M., Badhe S., Zhang J., Cantrell D.R., Tanhehco E.J., Szrama N., Naidech A.M., Drakopoulos M., Hasan S.T. (2024). Precise Image-level Localization of Intracranial Hemorrhage on Head CT Scans with Deep Learning Models Trained on Study-level Labels. Radiol. Artif. Intell..

[89] Ye H., Gao F., Yin Y., Guo D., Zhao P., Lu Y., Wang X., Bai J., Cao K., Song Q. (2019). Precise diagnosis of intracranial hemorrhage and subtypes using a three-dimensional joint convolutional and recurrent neural network. Eur. Radiol..

[90] Yedavalli V., Heit J.J., Dehkharghani S., Haerian H., Mcmenamy J., Honce J., Timpone V.M., Harnain C., Kesselman A., Filly A. (2023). Performance of RAPID noncontrast CT stroke platform in large vessel occlusion and intracranial hemorrhage detection. Front. Neurol..

[91] Yeo M., Tahayori B., Kok H.K., Maingard J., Kutaiba N., Russell J., Thijs V., Jhamb A., Chandra R.V., Brooks M. (2023). Evaluation of techniques to improve a deep learning algorithm for the automatic detection of intracranial haemorrhage on CT head imaging. Eur. Radiol. Exp..

[92] Yun T.J., Choi J.W., Han M., Jung W.S., Choi S.H., Yoo R.-E., Hwang I.P. (2023). Deep learning based automatic detection algorithm for acute intracranial haemorrhage: A pivotal randomized clinical trial. NPJ Digit. Med..

[93] Zhou Q., Zhu W., Li F., Yuan M., Zheng L., Liu X. (2022). Transfer Learning of the ResNet-18 and DenseNet-121 Model Used to Diagnose Intracranial Hemorrhage in CT Scanning. Curr. Pharm. Des..

[94] Zia A., Fletcher C., Bigwood S., Ratnakanthan P., Seah J., Lee R., Kavnoudias H., Law M. (2022). Retrospective analysis and prospective validation of an AI-based software for intracranial haemorrhage detection at a high-volume trauma centre. Sci. Rep..

[95] Bark D., Basu J., Toumpanakis D., Nyberg J.B., Bjerner T., Rostami E., Fällmar D. (2024). Clinical Impact of an AI Decision Support System for Detection of Intracranial Hemorrhage in CT Scans. Neurotrauma Rep..

[96] Rymer M.M. (2011). Hemorrhagic stroke: Intracerebral hemorrhage. Mo. Med..

[97] Elliott J., Smith M. (2010). The acute management of intracerebral hemorrhage: A clinical review. Anesth. Analg..

[98] Warman R., Warman A., Warman P., Degnan A., Blickman J., Chowdhary V., Dash D., Sangal R., Vadhan J., Bueso T. (2022). Deep Learning System Boosts Radiologist Detection of Intracranial Hemorrhage. Cureus.

[99] Watanabe Y., Tanaka T., Nishida A., Takahashi H., Fujiwara M., Fujiwara T., Arisawa A., Yano H., Tomiyama N., Nakamura H. (2021). Improvement of the diagnostic accuracy for intracranial haemorrhage using deep learning-based computer-assisted detection. Neuroradiology.

[100] Chen W., Zhu W., Kovanlikaya I., Kovanlikaya A., Liu T., Wang S., Salustri C., Wang Y. (2014). Intracranial calcifications and hemorrhages: Characterization with quantitative susceptibility mapping. Radiology.

