# Supplementary material for: Diagnostic Accuracy of Deep Learning for Intracranial Hemorrhage Detection in Non-Contrast Brain CT Scans: A Systematic Review and Meta-Analysis
Source: J Clin Med. 2025 Mar 30;14(7):2377. doi: 10.3390/jcm14072377 (PMC11989428; doi:10.3390/jcm14072377)
Supplement: Supplementary file 1 [file jcm-14-02377-s001.zip › jcm-3539338-supplementary.pdf]

Supplementary file

Table S1. Quality Assessment of Diagnostic Accuracy Studies-2 (QUADS-2) risk of bias assessment.

Study	Risk of bias				Applicability		
	Patient selection	Index test	Reference standard	Flow and timing	Patient selection	Index test	Reference standard
Abrigo 2023	Low	Low	Low	Low	Low	Low	Low
Alis 2022	Low	Low	Low	Low	Low	Low	Low
Altuve 2022	?	Low	?	?	Low	Low	Low
Angkurawaranon 2023	Low	Low	Low	Low	Low	Low	Low
Arbabshirani 2017	?	Low	Low	Low	Low	Low	Low
Arman 2023	?	Low	Low	Low	?	Low	Low
Babu 2024	?	Low	Low	Low	?	Low	Low
Bark 2024	Low	Low	Low	Low	Low	Low	Low
Buls 2020	Low	Low	Low	Low	Low	Low	Low
Chang 2018	?	Low	Low	Low	?	Low	Low
Chien 2022	Low	Low	?	Low	Low	Low	Low
Chilamkurthy 2018	Low	Low	Low	Low	Low	Low	Low
Cho 2019	Low	Low	Low	Low	Low	Low	Low
Choi 2024	Low	Low	Low	Low	Low	Low	Low
Colasurdo 2022	Low	Low	Low	Low	Low	Low	Low
Coorens 2023	Low	Low	?	Low	Low	Low	Low
Cortés-Ferre 2023	Low	Low	Low	Low	Low	Low	Low
D'Angelo 2024	Low	Low	Low	Low	Low	Low	Low
Dawud 2019	Low	Low	?	Low	Low	Low	Low
Del Gaizo 2024	Low	Low	Low	Low	Low	Low	Low
Dyer 2021	Low	Low	Low	Low	Low	Low	Low
Eldaya 2022	Low	Low	Low	Low	Low	Low	Low
Gibson 2022	?	Low	Low	Low	Low	Low	Low
Ginat 2019	Low	Low	Low	Low	Low	Low	Low
Ginat 2021	Low	Low	?	Low	Low	Low	Low
Gruschwitz 2021	Low	Low	Low	Low	Low	Low	Low
Guo 2022	Low	Low	Low	Low	Low	Low	Low
He 2024	?	Low	Low	Low	Low	Low	Low
Heit 2021	Low	Low	Low	Low	Low	Low	Low
Hofmeijer 2023	Low	Low	Low	Low	Low	Low	Low
Hopkins 2022	Low	Low	Low	Low	Low	Low	Low
Hu 2023	Low	Low	?	Low	Low	Low	Low
Kang 2023	Low	Low	Low	Low	Low	Low	Low
Kau 2021	Low	Low	Low	Low	Low	Low	Low
Kiefer 2024	Low	Low	Low	Low	Low	Low	Low

Kumaravel 2020	?	Low	Low	Low	?	Low	Low
Kundisch 2021	Low	Low	Low	Low	Low	Low	Low
Kuo 2019	Low	Low	Low	Low	Low	Low	Low
Lee 2018	Low	Low	Low	Low	Low	Low	Low
Lee 2020	Low	Low	Low	Low	Low	Low	Low
López-Pérez 2022	?	Low	Low	Low	Low	Low	Low
Majumdar 2018	Low	Low	Low	Low	Low	Low	Low
McLouth 2021	?	Low	Low	Low	?	Low	Low
Monteiro 2020	?	Low	Low	Low	Low	Low	Low
Nada 2024	Low	Low	Low	Low	Low	Low	Low
Neves 2023	Low	Low	Low	Low	Low	Low	Low
Nishi 2021	Low	Low	Low	Low	Low	Low	Low
Pettet 2024	Low	Low	Low	Low	Low	Low	Low
Phaphuangwittayakul 2022	Low	Low	?	Low	Low	Low	Low
Rao 2022	Low	Low	Low	Low	Low	Low	Low
Roshan 2024	Low	Low	Low	Low	Low	Low	Low
Salehinejad 2021	Low	Low	?	?	Low	Low	Low
Savage 2024	Low	Low	Low	Low	Low	Low	Low
Schmitt 2021	?	?	Low	Low	Low	Low	Low
Seyam 2022	Low	Low	Low	Low	Low	Low	Low
Sindhura 2023	?	Low	Low	Low	Low	Low	Low
Sreekrishnan 2023	Low	Low	Low	Low	Low	Low	Low
Teneggi 2024	Low	Low	Low	Low	Low	Low	Low
Thanellas 2023	Low	Low	?	Low	Low	Low	Low
Tharek 2022	?	Low	?	Low	Low	Low	Low
Trang 2024	Low	Low	Low	Low	Low	Low	Low
Villringer 2024	Low	Low	Low	Low	Low	Low	Low
Voter 2021	Low	Low	Low	Low	Low	Low	Low
Wang 2021	?	Low	?	Low	Low	Low	Low
Wang 2023	Low	Low	Low	Low	Low	Low	Low
Wang 2024	Low	Low	Low	Low	Low	Low	Low
Wu 2024	Low	Low	Low	Low	Low	Low	Low
Ye 2019	Low	Low	Low	Low	Low	Low	Low
Yedavalli 2023	Low	Low	Low	Low	Low	Low	Low
Yeo 2023	?	Low	Low	Low	Low	Low	Low
Yun 2023	Low	Low	Low	Low	Low	Low	Low
Zhou 2022	Low	Low	Low	Low	Low	Low	Low
Zia 2022	Low	Low	Low	Low	Low	Low	Low

? = Unclear

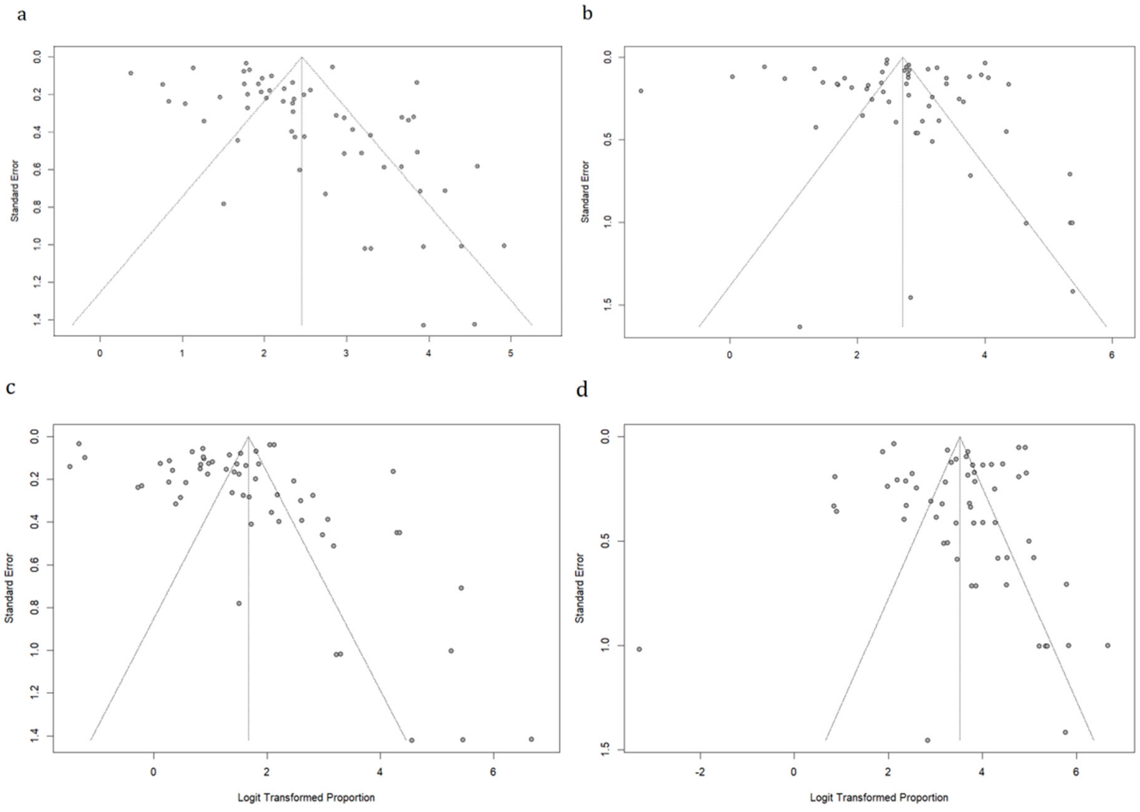

Figure S1. Funnel plots of sensitivity (a), specificity (b), PPV (c), and NPV (d).
